# Struggling toward reward: Recent experience of anhedonia interacts with motivation to predict reward pursuit in the face of a stressful manipulation

**DOI:** 10.1371/journal.pone.0173439

**Published:** 2017-03-08

**Authors:** Jessica Bryant, E. Samuel Winer, Taban Salem, Michael R. Nadorff

**Affiliations:** 1 Department of Psychology, Mississippi State University, Starkville, MS, United States of America; 2 Menninger Department of Psychiatry & Behavioral Sciences, Baylor College of Medicine, Houston, TX, United States of America; Universiteit Gent, BELGIUM

## Abstract

Anhedonia, or the loss of interest and/or pleasure, is a core symptom of depression. Individuals experiencing anhedonia have difficulty motivating themselves to pursue rewarding stimuli, which can result in dysfunction. Action orientation is a motivational factor that might interact with anhedonia to potentially buffer against this dysfunction, as action-oriented individuals upregulate positive affect to quickly motivate themselves to complete goals in the face of stress. The Effort-Expenditure for Rewards Task (EEfRT) is a promising new method for examining differences in motivation in individuals experiencing anhedonia. In the EEfRT, participants choose either easier tasks associated with smaller monetary rewards or harder tasks associated with larger monetary rewards. We examined the relationship between action orientation and EEfRT performance following a negative mood induction in a sample with varying levels of anhedonia. There were two competing hypotheses: (1) action orientation would act as a buffer against anhedonia such that action-oriented individuals, regardless of anhedonic symptoms, would be motivated to pursue greater rewards despite stress, or (2) anhedonia would act as a debilitating factor such that individuals with elevated anhedonic symptoms, regardless of action orientation, would not pursue greater rewards. We examined these hypotheses via Generalized Estimating Equations and found an interaction between anhedonia and action orientation. At low levels of anhedonia, action orientation was associated with effort for reward, but this relationship was not present at high levels of anhedonia. Thus, at low levels of anhedonia, action orientation acted as a buffer against stress, but at high levels, anhedonia debilitated action orientation so that it was no longer a promotive factor.

## Introduction

Anhedonia is a core symptom of depression [1–5]. Individuals experiencing anhedonia can have difficulty motivating themselves to pursue rewarding stimuli, thus causing dysfunction. Recent changes in anhedonia—that is, the *loss* of interest or pleasure—may be especially associated with depression [6–8]. This is because a loss signals that a person previously enjoyed and/or had interest in certain activities or social settings (i.e., had a functioning reward system), but that he or she is now getting less reward from those experiences [9,10]. As a result of reduced interest in or pleasure derived from activities once enjoyed, individuals experiencing anhedonia also experience lowered levels of positive affect. Positive affect plays an important role in volition, or the will to execute one’s goals [11], suggesting that the decreased levels of motivation seen in anhedonic individuals may result from a decrease in positive affect.

### Action and state orientation

Few studies have examined underlying motivational factors that might either predispose or protect an individual who is losing interest and/or pleasure from experiencing subsequent depression-related dysfunction. One candidate variable that might interact with recent changes in anhedonia is action orientation [11]. Action orientation is generally defined as high scores on the Action Control Scale [12], whereas state orientation is defined as low scores on the Action Control Scale. Action (state) orientation, for the purposes of the present study, refers to one’s ability (inability) to upregulate positive affect in order to pursue goals, especially in situations in which there is low positive affect or high negative affect.

Action-oriented individuals are able to upregulate positive affect in order to quickly move past obstacles or difficulties, whereas state-oriented individuals struggle to upregulate positive affect and are thus often unable to act efficiently [11,13]. Because of this inability to act, state-oriented individuals are more likely to be characterized as frustrated, hesitant, or indecisive than action-oriented individuals and are more likely to procrastinate when faced with difficult tasks or situations, rather than facing challenges outright [14–16].

How much one pauses before taking action to pursue a goal is a robust and predictive individual difference [11,17]. Moreover, these differences are clearest under conditions of stress. In low-stress situations state-oriented individuals will often complete tasks with ease and may even outperform action-oriented individuals (e.g., [18]). However, in stressful situations where there are higher levels of negative affect and upregulation of positive affect is necessary for goal pursuit, state-oriented individuals tend to perform poorly whereas action-oriented individuals excel [19]. Action control is a phenomenon that has also been investigated behaviorally, in conjunction with a mood induction procedure and affective priming task [20]. As part of this protocol, participants underwent a mood induction procedure where they were asked to visualize a demanding time in their life, and then were presented with primes of either negatively or positively valenced words before quickly determining the valence of a target word presented after the prime. Typically, when a prime is the same valence as the target, reaction times are shorter; but, when the prime and target are different valences, reaction times are longer. However, Koole and Fockenberg [20] predicted that those who were action-oriented would display weaker negative affective priming than state-oriented individuals. Action-oriented individuals would be more efficient, such that under stress, the negative primes would trigger down-regulation of the negative emotion. As predicted, the impact of action and state orientation on affective priming was moderated by situational demands. Action-oriented individuals presented with negative primes displayed reversed affective priming (i.e., when provided with valence-congruent prime and target–an example would be a positive prime and a positive target–the reaction times would be longer rather than shorter, the opposite of the normal affective priming pattern) in comparison to state-oriented individuals, who displayed standard congruency effects. These findings suggest that action-oriented individuals were more readily able to downregulate negative affect, whereas state-oriented individuals were not as able to downregulate negative affect.

Thus, there is evidence to suggest that the tendency to be action-oriented enables a person to pursue rewards even in the face of high levels of negative affect or low levels of positive affect. By extension, it is possible that action orientation might offset or buffer against anhedonia, such that action-oriented individuals experiencing loss of interest and/or pleasure would be able to upregulate positive affect in the presence of low levels of positive affect to approach rewards. Conversely, it is possible that self-reported recent loss of interest and/or pleasure might be debilitating on a motivational level and therefore be associated with a lack of reward pursuit regardless of a person’s usual tendency to be either action- or state-oriented. Lastly, anhedonia and action control might interact such that individuals recently experiencing high levels of anhedonia would not benefit from being action-oriented, but action-oriented individuals with milder anhedonic symptoms would still be able to pursue rewards, even in the face of stress.

### Examining the pursuit of reward

The Effort-Expenditure for Rewards Task (EEfRT) recently introduced by Treadway and colleagues [21] is an ideal task for examining reward pursuit. The task was adapted from the concurrent choice paradigm used to assess reward pursuit with rodents [22], and it examines the multi-faceted reward decision-making process, with particular focus on perseverance and cost-benefit analysis of reward.

In each trial of the EEfRT, participants are given the choice between attempting a hard task for the chance to win a larger monetary prize (high cost/high reward trial; HC/HR) or an easy task for the chance to win a smaller monetary prize (low cost/low reward trial; LC/LR). To help them decide, they are told the reward amount associated with each option and the probability that the given trial will be a “win” trial (i.e., one in which they may be eligible to receive the money if they successfully complete the trial). They are then given five seconds to decide whether to pursue the HC/HR trial or the LC/LR trial. If they are unable to make a decision within that time, then the computer program chooses the trial for them at random. Also, participants are informed at the beginning of the EEfRT that they will have 20 minutes to complete as many trials as they can, and that the HC/HR trials are more time-consuming, so choosing this option reduces the number of trials that can be completed within the time limit.

Thus, the EEfRT provides an objective means by which to investigate reward pursuit, beyond that of traditional self-report measures of anhedonic symptoms. Among the EEfRT’s strengths is its ability to disentangle multiple aspects of the reward-pursuit process, yielding relatively specific indexes of perseverance, cost-benefit-analysis, and pursuit of reward based on its potential magnitude. Using the EEfRT, Treadway and colleagues found that individuals with depressive symptoms were less willing to exert effort to obtain a larger monetary reward than were healthy controls [23]. Individuals with depressive symptoms also showed less sensitivity to information regarding reward magnitude and probability that the trial would be a ‘win’ trial when deciding which trial to pursue (i.e., a HC/HR or LC/LR task).

Recently, Geaney and colleagues [24] used the EEfRT in conjunction with a positive mood induction and a self-report measure of anhedonia (the Temporal Experience of Pleasure Scale; TEPS, [25]) to assess the relationship between level of anhedonia, positive mood, and motivation for reward. Their study found that level of anticipatory anhedonia predicted pursuit of reward when probability of reward receipt was low, such that individuals experiencing low levels of anticipatory anhedonia pursued reward more often, but this relationship was not significant when probability was at medium or high levels. However, interactions between level of anticipatory anhedonia with reward amount and expected value (reward amount x probability) respectively were not predictive of reward pursuit. Also, levels of consummatory and anticipatory anhedonia alone and positive affect alone did not predict reward pursuit.

Geaney and colleagues [24] provided answers to a number of important questions regarding anhedonia and motivation under conditions of positive affect. However, their study did not investigate the effects of anhedonia on the level of effort for reward in the face of negative affect. A study examining anhedonia and reward pursuit following a negative mood manipulation would bridge this gap in the literature. A negative mood manipulation would also be ideal for examining anhedonia and reward pursuit in relation to motivational qualities such as action control, as differences between action- and state-oriented individuals are clearest when they are confronted with a stressful situation [19]. When state-oriented individuals are not faced with stressful situations that require upregulation of positive affect to complete a goal, they will often not find tasks difficult, and may even outperform action-oriented individuals (e.g., [18]). Additionally, previous studies have generally investigated anhedonia and reward pursuit using trait-based anhedonia measures. However, evidence suggests that using a measure of anhedonia that can assess changes from baseline in individuals may provide added prediction of depressogenic processes that intrinsically ebb and flow [7,8]. Previous measures have not included wording that captures a change from the norm, and thus may inadvertently capture trait-based responses. Previously, Joiner and colleagues [26] attempted to address this gap in the literature through use of selected items related to anhedonia from the Beck Depression Inventory. Only recently was a validated measure available to assess for changes in experience of anhedonia rather than a subset of items from another questionnaire, however [7].

### Current study and hypotheses

In summary, action- and state-oriented individuals differ in their ability to upregulate positive affect when presented with demanding or stressful situations [18–20]. In addition, people with recent changes in anhedonia are currently experiencing less positive affect than what is normal for them, and are more likely to be at risk for depression and suicidal ideation in the near future [7,8]. Self-reported anhedonia is negatively associated with perseverance in demanding situations, as demonstrated by the EEfRT [23]. Additionally, low levels of anhedonia predict higher levels of reward pursuit when probability of reward receipt is low [24].

However, when examining individual predictors of reward pursuit, level of anhedonia or level of positive affect alone are not predictive [24], and the manner in which recent changes in anhedonia and action orientation interact to predict reward pursuit is currently unknown. It may be that anhedonic individuals are so often state-oriented that they have chronic difficulty upregulating positive affect in the face of stress, and thus motivating themselves toward rewarding goals. Conversely, it may be that having an action-oriented motivational tendency provides a buffer in the face of limited positive affect, such that action-oriented individuals continue to persevere in the face of stress even when they are less motivated to do so by other motivational systems [11,27,28]. Also, to date there has been no investigation of the relationship between anhedonia, action control, and reward pursuit with a negative mood manipulation included. Therefore, the purpose of the present study is to bridge these gaps in the literature by examining the interaction of recent changes in anhedonia and action orientation in relation to the pursuit of reward on the EEfRT, in the face of a demanding mood induction.

Two competing hypotheses were investigated. The ‘buffering hypothesis’ predicted that motivation and anhedonia would interact, such that action orientation would act as a buffer against low levels of positive affect for those experiencing anhedonia, allowing these individuals to pursue rewards in the EEfRT. The ‘debilitation hypothesis’ posited that anhedonia would be debilitating on a motivational level, such that action orientation would not buffer against the effects of recent changes in anhedonia in a demanding situation, and thus there would be no interaction between motivation and anhedonia with regard to EEfRT performance.

## Materials and methods

### Ethics statement

The Mississippi State University Institutional Review Board approved this research (#14–247). Participants provided written consent for the study. Participants were initially informed that they were taking part in two separate, unrelated studies to guard against demand characteristics. Participants were fully debriefed at the conclusion of data collection regarding the fact that they were told that they participated in two studies, but actually only participated in one study. Written consent was recorded via two informed consent documents, and consent and debriefing procedures were approved by the IRB.

### Participants

Seventy-six participants (54.3% female, ages 18–56, 77.1% Caucasian, 11.4% African American, 11.5% other) from Mississippi State University were recruited online through the university’s undergraduate research pool to take part in the study (recruitment occurring from September to November 2014). Students in the undergraduate research pool were given one month to complete prescreening questions online via the SONA system. After one month, the responses of participants who had completed the screener were examined and used for pre-selection for the present study. Specifically, the Temporal Experience of Pleasure Scale (TEPS; [25]) and the decision-related action orientation versus hesitation (AOD) subscale from the Action Control Scale (ACS; [12]) were used to pre-select participants. The TEPS measures anticipatory and consummatory pleasure, and the AOD subscale measures action/state orientation as it applies to upregulation of positive affect to make decisions or take action toward goals. Participants were selected who were either in the top (scores ranging from 9–12) or bottom (scores ranging from 1–5) quartiles of the AOD subscale (highly action- and highly state-oriented, respectively). All of these participants were also in the bottom quartile (scores ranging from 1–44) of the anticipatory subscale of the TEPS (high deficit in anticipatory pleasure) to ensure that participants had high enough symptoms of anhedonia.

Individuals who met pre-selection criteria were sent an email via the online SONA system inviting them to participate in the study. Participants received course credit for their time and participation, as well as a variable sum of money ranging from $2.00 to $8.60 as a bonus for their performance on the EEfRT. Participants were excluded from final analyses for reporting a past diagnosis of bipolar disorder or psychosis (1 participant; this indicated the potential that a participant may have a severe mental illness, which we were not attempting to capture within this sample), not properly completing the computerized task (2 participants; described in the data screening section), for ambidexterity (1 participant), not being age 18 or older (1 participant), and for missing data due to camera malfunctions (1 participant). Participants were also assessed for physical disabilities that would have prevented them from completing the study, ability to speak or read Chinese (which would have interfered with one of the manipulation checks), and vision issues that would have prevented them from being able to read the computer screens, but no participants were excluded based on these factors.

### Measures

#### Action versus state orientation

Action and state orientation were assessed using the Action Control Scale (ACS, [12]). The ACS is made up of three subscales: failure-related action orientation versus preoccupation (AOF), decision-related action orientation versus hesitation (AOD), and performance-related action orientation versus volatility (AOP). In this study, the AOD subscale was used because of its prospective relation to upregulation of positive affect and decision-making [11,13]. This subscale is composed of 12 items and demonstrated adequate internal consistency (current study, α = .79; original paper, α = .78). Each item describes a scenario and two alternative answer choices. For example, one of the scenarios is “When I must finish something soon…” and the answer choices are “I have to push myself to get started,” which is indicative of state orientation, or “I find it easy to get it done and over with,” which is indicative of action orientation.

#### Recent changes in anhedonia

Recent changes in anhedonia were assessed using the Specific Loss of Interest and Pleasure Scale (SLIPS, [7]). The SLIPS is a 23-item self-report measure of changes in anhedonia in reference to the past two weeks. The scale demonstrated adequate internal consistency (current study, α = .89; original paper, α = .94). Each item on the SLIPS has four possible options, rated 0–3. For example, “I still enjoy going out with friends” (no change) would be a zero, “I don’t enjoy going out with friends as much as I used to” (slight change) would be a one, “I no longer enjoy going out with friends” (substantial change) would be a two, and “I have never enjoyed going out with anyone” (trait anhedonia) would be a three. Responses of one and two on the SLIPS are indicative of recent changes in anhedonia, which can be uniquely predictive of psychological crises [8]. The SLIPS allows for responses of three to be recoded as zero to assess only recent changes in anhedonia without including trait anhedonia [7]. This study followed the standard recoding procedure such that trait responses (scores of 3) were recoded as zero. The SLIPS was used for this study as it does include the unique ability to assess for recent changes rather than a trait-like or more long-term anhedonic presentation.

#### Motivation for reward

The Effort-Expenditure for Rewards Task (EEfRT, [21]) is a computerized task that assesses motivation for reward. The task evaluates several facets of reward motivation, such as probability of reward (i.e., the likelihood that a particular trial will be a ‘win’ trial in which a participant is eligible to receive money), choice of task difficulty in relation to prospective reward (high cost, high reward versus low cost, low reward), and effort toward obtaining reward. For the LC/LR, or “easy” task, participants were instructed to execute 30 button presses with the index finger of their dominant hand within seven seconds, and were eligible to win a consistent amount ($1.00) on each LC/LR trial that was completed. For the HC/HR, or “hard” task, participants were instructed to execute 100 button presses with the pinky finger of their non-dominant hand within 30 seconds, and were eligible to win larger amounts that varied from trial to trial, ranging from $1.24-$4.30. Participants were not guaranteed to win money for completing a trial; some trials were “win” trials for which the participant was eligible to receive the money, and others were “no win” trials for which the participant was not eligible to receive the money. At the end of the EEfRT, two “win” trials were selected at random and participants received the amount of money that they won in those two trials. In each trial before choosing the LC/LR or HC/HR option, participants were told the probability, 88% (high), 50% (medium), or 12% (low), that the upcoming trial would be a “win” trial if successfully completed. Across the trials there were equal numbers of each reward probability (88%, 50%, and 12%), so no single probability level was presented more than another.

### Mood induction

#### Procedure

Following Koole and Fockenberg [20], participants were asked to mentally visualize a particularly demanding time in their life. During visualization, participants were asked to try to recall their thoughts and feelings from that time, and to attempt to re-experience the situation by recalling this information. Participants were also asked to write down some of the thoughts and feelings that were related to the situation they chose.

#### Manipulation checks

The Positive and Negative Affect Schedule (PANAS, [29]) was used to assess the effectiveness of the mood manipulation. The PANAS is a 20-item self-report measure that consists of 10 positive words and 10 negative words. Participants are asked to rate on a scale of one (very slightly or not at all) to five (extremely) to what extent they are currently identifying with each word. Both the positive and negative affect subscales show adequate internal consistency (current study, α = .78 and .80; previous work, α = .89 and .85, respectively).

A previously unvalidated brief implicit measure of emotion [30] was also used to examine if the mood manipulation was effective. This six-item implicit computerized measure assesses the experience of specific emotional states that may be outside of conscious awareness or less likely to be endorsed [31]. For each item, participants were presented with a Chinese character on the computer and asked to select the emotion (anger, fear, happiness, sadness, or no emotion) to which they believed the character corresponded. For pre- and post-manipulation scores, counts for each mood state (anger, fear, happiness, sadness, no emotion) were taken. To obtain a change score, pre scores were subtracted from post scores.

Participants were given both the implicit measure of distinct emotions and the PANAS prior to the mood induction to assess baseline mood. After the induction, participants were given the measures again to assess change from baseline.

### Procedure

Participants completed an informed consent process, which consisted of two informed consent documents, provided by two separate experimenters when the participant initially arrived in the laboratory. Participants were told that the protocol consisted of two unrelated studies, each with its own consent document. Experimenters were blind to the study hypotheses, and one experimenter ran each “study.” Deception was used in order to minimize the likelihood of demand characteristics that could arise if participants were to connect the mood induction with their EEfRT performance. Participants completed the self-report measures assessing action control (ACS) and recent changes in anhedonia (SLIPS), as well as other questionnaires related to various research questions. All measures were presented on computer via E-Prime, Version 2.0. Participants then underwent a mood induction, detailed above. Manipulation checks were administered before and after the induction. Participants then completed the EEfRT [21], which was given on the computer. Before beginning the EEfRT, participants were informed by their experimenter that they were being video recorded to ensure that they were using the correct fingers during the HC/HR and LC/LR trials. During the task, the first experimenter watched the video (recorded via Edimax IC-3110W IP camera and streamed via Ethernet to a computer in a conjoining room) in real time and coded each trial for finger accuracy in an Excel sheet. A second experimenter later examined only the video trials that the first experimenter reported were performed incorrectly to confirm trial inaccuracy. After the EEfRT, participants completed additional questionnaires. Lastly, participants were paid, provided with referral information for available psychological services, and credited for their participation. The experimental procedure took between 1–1.5 hours in total. Participants were debriefed at the conclusion of data collection.

## Results

### Data screening

One participant had missing data for the pre-manipulation PANAS and the implicit measure of distinct emotions due to experimenter error. That participant was not included in the manipulation check analyses, but was included for all other analyses. Correlations between the SLIPS and AOD were computed to assess for overlap. The two measures correlated slightly (*r* = -.32). The mean of the SLIPS for our sample was 8.23, (*SD* = 7.28) whereas the mean in the original paper was 9.27. Thus, for our sample, the mean was slightly lower, but was still within the range of the original paper. The mean of the AOD subscale was 6.11, and the standard deviation was 3.13. For the EEfRT, video recordings were checked by two independent coders, and trials on which participants did not properly complete the task (i.e., did not use the correct fingers for trials or used one hand to push the other to complete trials) were removed from data analysis. Participants were excluded if more than 10% of trials were performed incorrectly, resulting in the removal of two participants for incorrectly completing the task. Additionally, the effects of incomplete trials were assessed by running Model 1 (shown below) with completion of trial as an additional variable and, although significant, this addition did not affect the significance of any of the critical variables. For the current analyses, the first 50 trials completed by each participant were used for consistency [21].

### Manipulation check

#### PANAS

Two paired-samples *t*-tests were conducted to compare the pre- and post-manipulation scores for the Positive Affect (PA) and Negative Affect (NA) PANAS subscales. The pre- (*M* = 17.68, *SD* = 6.98) and post-manipulation (*M* = 17.00, *SD* = 7.74) scores for the NA subscale did not significantly differ, *t*(68) = 1.86, *p* = .067, *d* = .22. However, there was a significant difference in the pre- (*M* = 29.28, *SD* = 6.41) and post-manipulation (*M* = 27.41, *SD* = 8.32) scores for the PA subscale, *t*(68) = 3.45, *p* = .001, *d* = .42. Thus, although participants did not report a significant change in their level of negative affect after the mood induction, they did report feeling significantly less positive affect after the induction. To supplement this finding, we examined two key items on the PANAS NA subscale: item 2 (distressed) and item 4 (upset). Two paired-samples *t*-tests were used to examine the differences in the pre- and post-manipulation scores on these items. For “distressed,” there was no significant difference in the pre- (*M* = 1.99, *SD* = 1.23) and the post-manipulation *(M* = 1.87, *SD* = 1.04) scores, *t*(68) = 1.16, *p* = .251, *d* = .14. However, there was a significant difference in the predicted direction between the pre- (*M* = 1.45, *SD* = .98) and the post-manipulation (*M* = 1.78, *SD* = 1.07) scores for “upset,” *t*(68) = -2.96, *p* = .004, *d* = .36. Even though there was no significant change in the pre- and post-manipulation scores for the NA subscale as a whole, participants did self-report feeling more upset after the manipulation. These results provide evidence that the mood induction manipulation had the desired effect of decreasing participants’ positive affect and invoking an upsetting experience, which is buttressed by the following results from the implicit manipulation check.

#### Brief implicit measure of distinct emotions

Change scores were entered into a MANOVA to test for changes in how frequently each of the mood states was selected. There were two significant changes from pre- to post- manipulation: sadness (*M* = .19, *F*(1, 69) = 5.11, *p* = .027, partial eta squared = .07), such that individuals selected sadness more frequently after the induction, and no emotion (*M* = -.23, *F*(1, 69) = 4.33, *p* = .041, partial eta squared = .06), such that individuals selected no emotion less often after the induction in comparison to baseline. No significant changes were found for anger, fear, or happiness. These findings suggest that participants were likely feeling less positive and more sad and upset following the mood manipulation.

### Main analyses

Data were analyzed using four generalized estimating equations (GEE) using SPSS Version 23. GEE is the primary analytic technique used with EEfRT data, because as a generalized regression model it allows nested investigation of data that are interdependent, and thus have an unknown level of correlation with one another. GEE is ideal for EEfRT data, as many of the EEfRT variables are taken within a single trial and thus are intercorrelated to a degree [32]. GEE models allow for modeling of trial-level changes (such as changes in reward amount or probability) and also between-subjects differences, such as level of anhedonia or action control. As these models focus on the trial level rather than the subject level, GEE models increase statistical power.

Following the analytic approach of previous EEfRT investigations [21,33], the dependent variable in all models was dichotomous HC/HR or LC/LR task choice, and a binary logistic distribution was used to model the probability of choosing the HC/HR option. As a binary logistic distribution was used, only responses indicating if the participant chose the HC/HR or LC/LR task were included. Trials for which participants did not choose either task within the time limit were not included in analyses; altogether this amounted to 1.1% missing data, which is within the 5% limit Tabachnick and Fidell [34] outline for using any method of handling missing data. All models used an unstructured working correlation matrix and included probability, reward amount, and expected value (calculated as probability x reward amount) as independent variables. Additionally, each model included the interaction of anhedonia (measured by the SLIPS) and action control (measured by the ACS-AOD subscale).

Model 1 (QIC = 3779.21) assessed main effects of sex, trial number, probability, reward amount, expected value, anhedonia, action control, and the interaction of anhedonia and action control. Higher expected value significantly predicted higher HC/HR choice (*b* = .995, *p* = .005), as did anhedonia (*b =* .070, *p* < .05), and action control (*b =* .088, *p* < .05), but reward amount and probability were not significant predictors of task choice. Interestingly, anhedonia evidenced a positive association with HC/HR choice. However, these main effects were qualified by the interaction of anhedonia and action control, which was found to be a significant predictor of HC/HR choice (*b* = -.009, *p* < .05).

**Fig 1:**
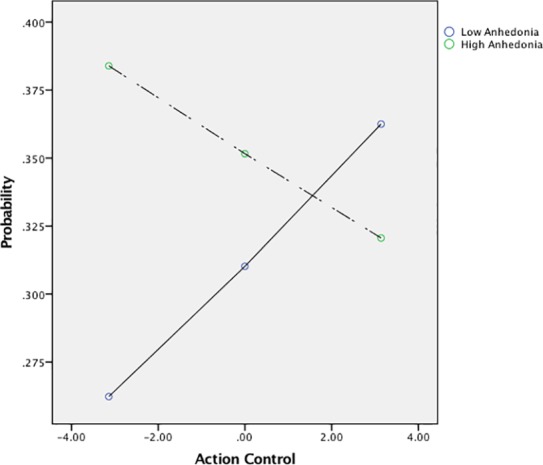
Graph depicting the positive relationship between action control and effort when anhedonia is low. *Note*. This figure represents unique simple slopes from a regression-based PROCESS analysis that creates plotted values. These slopes are comparable to those represented in Model 1, but do not contain all variables of the GEE analysis.

Follow-up analyses were performed to probe the interaction of anhedonia and action control. Interaction terms were constructed at high and low levels of anhedonia (+/- 1 *SD*). This analysis revealed that action control was associated with HC/HR choice at low levels of anhedonia (*b =* .080, *p* = .039), but not at high levels of anhedonia (*b* = -.050, *ns*). Please see Fig 1.

Model 2 (QIC = 3787.83) examined the three-way interaction of probability, anhedonia, and action control, which was not significant (*b =* .002, *ns*). Model 3 (QIC = 3784.00) examined the three-way interaction of reward amount, anhedonia, and action control. Again, there was no evidence for this interaction (*b =* .002, *ns*). Model 4 (QIC = 3782.92) assessed the three-way interaction between expected value, anhedonia, and action control. No evidence was found for this interaction as well (*b =* .003, *ns*). Please see Table 1 for full breakdown of the four GEE models. For all models, trial number was significant, indicating that, over time, participants became more fatigued, which is a common finding when using the EEfRT [21].

**Table 1:** GEE Models 1–4.

		*b*	*SE*	*p*	95% LCI	95% UCI
Model 1						
	Sex	.033	.1807	.857	-.322	.387
	Trial Number	-.015	.0032	< .001	-.022	-.009
	Probability	-.048	1.1016	.965	-2.207	2.111
	Reward Amount	.151	.1967	.443	-.235	.536
	Expected Value	.995	.3514	.005	.306	1.684
	SLIPS	.070	.0316	.026	.008	.132
	AOD	.088	.0417	.035	.006	.170
	SLIPS*AOD	-.009	.0042	.035	-.017	-.001
Model 2						
	Sex	.032	.1812	.861	-.323	.387
	Trial Number	-.015	.0033	< .001	-.022	-.009
	Probability	-.431	1.4173	.761	-3.209	2.347
	Reward Amount	.159	.1962	.418	-.226	.543
	Expected Value	.984	.3500	.005	.298	1.670
	SLIPS	.080	.0411	.052	-.001	.161
	AOD	.039	.0766	.608	-.111	.189
	SLIPS*AOD	-.010	.0068	.151	-.023	.004
	Probability*AOD	.085	.1258	.498	-.161	.332
	Probability*SLIPS	-.020	.0539	.705	-.126	.085
	Probability*SLIPS*AOD	.002	.0110	.874	-.020	.023
Model 3						
	Sex	.028	.1810	.876	-.326	.383
	Trial Number	-.015	.0032	< .001	-.022	-.009
	Probability	-.026	1.0806	.981	-2.144	2.092
	Reward Amount	.227	.3141	.469	-.388	.843
	Expected Value	.991	.3453	.004	.315	1.668
	SLIPS	.070	.0500	.161	-.028	.168
	AOD	.164	.0972	.091	-.026	.355
	SLIPS*AOD	-.015	.0084	.073	-.032	.001
	Reward Amount*AOD	-.028	.0328	.394	-.092	.036
	Reward Amount*SLIPS	.000	.0148	.979	-.029	.029
	Reward Amount*SLIPS*AOD	.002	.0026	.404	-.003	.007
Model 4						
	Sex	.027	.1813	.881	-.328	.382
	Trial Number	-.015	.0032	< .001	-.022	-.009
	Probability	.050	1.0736	.963	-2.054	2.154
	Reward Amount	.169	.1920	.379	-.208	.545
	Expected Value	.891	.4216	.035	.065	1.717
	SLIPS	.089	.0401	.027	.010	.167
	AOD	.076	.0645	.241	-.051	.202
	SLIPS*AOD	-.014	.0054	.010	-.024	-.003
	Expected Value*AOD	.007	.0368	.846	-.065	.079
	Expected Value *SLIPS	-.013	.0168	.425	-.046	.020
	Expected Value *SLIPS*AOD	.003	.0029	.256	-.002	.009

## Discussion

We hypothesized that action orientation would act in one of two ways in the presence of anhedonia: (1) action orientation would buffer against anhedonia in the face of stress, allowing individuals to pursue the HC/HR option on the EEfRT (the buffering hypothesis), or (2) action orientation would not buffer against anhedonia in the face of stress and anhedonia would be a debilitating factor, such that individuals would not pursue the HC/HR option (the debilitation hypothesis). Results of Model 1 provide evidence for both the debilitation hypothesis, as action control *was not* associated with HC/HR choice at *high* levels of anhedonia, and the buffering hypothesis, as action control *was* associated with HC/HR choice at *low* levels of anhedonia. This outcome supports the assertion that at high levels, anhedonia is debilitating even for action-oriented individuals, but at low levels of anhedonia, action orientation may act as a buffer against stress and therefore enable individuals to pursue rewarding stimuli. Our findings suggest that individuals who report a trait ability to upregulate positive affect in times of stress are in fact unable to do so when experiencing recent increases in anhedonic symptoms.

Previous research has found that chronic exposure to stressors may induce mild levels of anhedonia, which in turn decreases responsiveness to reward at a neurobiological level [35]. Additionally, increased sensitivity to stressors and difficulties with reward processing are factors that persist even after remission of other depressive symptoms. The relationship between stressors and dysfunctional reward response could potentially link to anhedonia and other depressive symptoms [36]. Thus, this provides evidence that action-oriented individuals, who are ‘energized’ by stressful situations, may indeed be buffered initially against the effects of mild stress and anhedonia. Stressors that are more chronic may lead to higher levels of anhedonia and may be debilitating rather than protective for these action-oriented individuals, consistent with our findings here.

Clinically, our findings are important because even individuals who are able to upregulate positive affect in times of high negative affect or low positive affect are unable to utilize action orientation when experiencing high levels of anhedonia to motivate themselves. Thus, high levels of anhedonia are debilitating at a motivational level, which may keep individuals from pursuing rewarding goals and therefore increase their dysfunction related to depression. If individuals are unable to motivate themselves to go out and pursue experiences or things that are rewarding, then this provides evidence for treatments such as behavioral activation. In this treatment, individuals with depression are asked to go out and pursue experiences or things that they once found rewarding. Clients are held accountable for completing these activities, which provides external motivation for those who may be unable to motivate themselves internally.

Additionally, the other finding that emerged from this study was that at low levels of anhedonia, action orientation acted as a buffer. This provides evidence that action orientation may be a protective factor against motivational deficits for individuals beginning to experience anhedonia, or that, as suggested above, over time chronic stressors may be more impairing. Thus, action-oriented individuals experiencing anhedonia may be less likely or may take longer to develop motivational deficits than their anhedonic, state-oriented counterparts.

### Strengths and limitations

There were several potential limitations to this study. First, the study used a student sample. Using student samples limits the generalizability of the results, as most participants in undergraduate subject pools are college freshmen enrolled in introductory psychology classes who may not be intrinsically motivated to participate in research. To combat this limitation and increase motivational spread, as in the original EEfRT paper [21] and in subsequent papers [23,33,37], participants were given an additional monetary incentive based partially on the amount of effort they exerted. Additionally, *we pre-selected participants based on both high and low action orientation and high levels of anticipatory anhedonia*, thus greatly increasing power for uncovering the interaction that emerged [38].

Another potential limitation is that we incorporated within-subject manipulation checks, which could have potentially led to demand characteristics. However, we did see the anticipated change in affective states after our manipulation, within both manipulation check measures. A notable strength offsetting this limitation was that the manipulation check included not only an explicit but also an implicit procedure, thus providing multifaceted support that the manipulation check was effective.

Information regarding participants’ use of psychiatric medications and medical or psychiatric history (aside from previous diagnosis of bipolar disorder or psychotic experiences) was not obtained. As psychiatric medications could have potentially affected the level of anhedonia that participants experienced, this is listed as a potential limitation to this work. However, this study focused on level of anhedonia as a cross-sectional variable, so this change in level of anhedonia does not affect our results.

Additionally, this study only included a negative mood induction, rather than including two separate mood inductions to assess both negative and positive mood states. However, as mentioned in the Introduction section of this paper, Geaney and colleagues [24] employed a positive mood induction in conjunction with the EEfRT and a measure of anhedonia in their recent study. Therefore, the effects of both positive and negative mood states can be assessed when considering the two studies together. Moreover, a within-subject manipulation incorporating both positive and negative mood inductions would have been unwieldly and would have increased the likelihood of demand characteristics, and a between-subject assessment would have potentially reduced power due to the pre-selection criteria for our individual difference variables.

A last notable strength of this study was the use of two separate experimenters to bolster the cover story that participants were involved in two separate studies and that the mood manipulation was unrelated to the EEfRT. This additional aspect helped to limit the transparency of the design, and may have contributed to the success of the mood manipulation and further allowed for the findings that emerged.

### Future directions and conclusion

Future research should examine the relationships between anhedonia, action control, and effort toward reward using longitudinal methods, so that any fluctuation in the relationships could be assessed over time. Using longitudinal methods would allow for temporal associations to be interpreted and potential causal mechanisms to be inferred, whereas the current cross-sectional design is limited in this regard [39]. Moreover, along with examining the EEfRT’s association with self-report measures over time, comparing the EEfRT to other candidate self-report measures and behavioral tasks [40–42] will provide a fuller picture of the relation between low reported positive affect, reward pursuit, and reward devaluation [10].

Taken together, the current study’s constellation of findings suggest that action orientation does buffer in the face of stress so that reward pursuit is not affected, but only when anhedonia has not recently increased. When individuals have recently experienced an increase in symptoms of anhedonia, those symptoms are debilitating at a motivational level, keeping them from pursuing rewarding goals regardless of action orientation and therefore potentially increasing their dysfunction related to depression.

## Supporting information

S1 Dataset Raw data file. (CSV)

## References

[1] Borsboom D., Cramer AOJ. Network analysis: An integrative approach to the structure of psychopathology. Annu Rev Clin Psychol. 2013;9:91–121. 10.1146/annurev-clinpsy-050212-185608 23537483

[2] Cramer A.O., Waldorp L.J., Van Der Maas H.L., Borsboom D. Comorbidity: A network perspective. Behav Brain Sci. 2010;33:137–93. 10.1017/S0140525X09991567 20584369

[3] Treadway MT, Zald DH. Reconsidering anhedonia in depression: Lessons from translational neuroscience. Neurosci Biobehav Rev. 2011;35(3):537–55. 10.1016/j.neubiorev.2010.06.006 20603146 PMC3005986

[4] Treadway MT, Zald DH. Parsing anhedonia: Translational models of reward-processing deficits in psychopathology. Curr Dir Psychol Sci. 2013;22(3):244–9. 10.1177/0963721412474460 24748727 PMC3989147

[5] Whitton A.E., Treadway M.T., Pizzagalli DA. Reward processing dysfunction in major depression, bipolar disorder and schizophrenia. Curr Opin Psychiatry. 2015;28:7–12. 10.1097/YCO.0000000000000122 25415499 PMC4277233

[6] Winer E.S., Drapeau C.W., Veilleux J.C., Nadorff MR. Anhedonia, suicidal ideation, and suicide attempts in a large student sample. Arch Suicide Res. 2016;20(2):265–72. 10.1080/13811118.2015.1025119 26214573

[7] Winer ES, Veilleux JC, Ginger EJ. Development and validation of the Specific Loss of Interest and Pleasure Scale (SLIPS). J Affect Disord. 2014;152-154(1):193–201. 24099883 10.1016/j.jad.2013.09.010

[8] Winer E.S., Nadorff M.R., Ellis T.E., Allen J.G., Herrera S., Salem T. Anhedonia predicts suicidal ideation in a large psychiatric inpatient sample. Psychiatry Res. 2014;218:124–8. 10.1016/j.psychres.2014.04.016 24774075

[9] Gotlib I.H., McLachlan A.L., Katz AN. Biases in visual attention in depressed and nondepressed individuals. Cogn Emot Issue Inf Process Emot Disord. 1988;2:185–200.

[10] Winer ES, Salem T. Reward devaluation: Dot-probe meta-analytic evidence of avoidance of positive information in depressed persons. Psychol Bull. 2016;142(1):18–78. 10.1037/bul0000022 26619211 PMC4688138

[11] Kuhl J, Koole SL. Workings of the will. Handb Exp Existent Psychol. 2004;411–30.

[12] Kuhl J. Action and state orientation: Psychometric properties of the action control scales (ACS-90) In: Kuhl J. & Beckmann J., editor. Volition and Personality: Action versus State Orientation. Göttingen, Germany: Hogrefe; 1994 p. 47–59.

[13] Kuhl J. The volitional basis of personality systems interaction theory: Applications in learning and treatment contexts. Int J Educ Res. 2000;33(7–8):665–703.

[14] Beckmann J. & Kuhl J. Altering information to gain action control: Functional aspects of human information processing in decision making. J Res Pers. 1984;18(2):224–37.

[15] Blunt A, Pychyl T a. Volitional action and inaction in the lives of undergraduate students: State orientation, procrastination and proneness to boredom. Pers Individ Dif. 1998;24(6):837–46.

[16] Diefendorff JM, Hall RJ, Lord RG, Strean ML. Action-state orientation: construct validity of a revised measure and its relationship to work-related variables. J Appl Psychol. 2000;85(2):250–63. 10783541 10.1037/0021-9010.85.2.250

[17] Kazén M, Kaschel R, Kuhl J. Individual differences in intention initiation under demanding conditions: Interactive effects of state vs. action orientation and enactment difficulty. J Res Pers. 2008;42(3):693–715.

[18] Jostmann NB, Koole SL. On the waxing and waning of working memory: action orientation moderates the impact of demanding relationship primes on working memory capacity. Pers Soc Psychol Bull. 2006;32(12):1716–28. 10.1177/0146167206292595 17122182

[19] Menec V. H., Perry R. P., & Struthers C. The effect of adverse learning conditions on action-oriented and state-oriented college students. J Exp Educ. 1995;63(4):281–99.

[20] Koole SL, Fockenberg D a. Implicit emotion regulation under demanding conditions: the moderating role of action versus state orientation. Cogn Emot. 2011;25(3):440–52. 10.1080/02699931.2010.544891 21432685

[21] Treadway MT, Buckholtz JW, Schwartzman AN, Lambert WE, Zald DH. Worth the “EEfRT”? The effort expenditure for rewards task as an objective measure of motivation and anhedonia. PLoS One. 2009;4(8):e6598 10.1371/journal.pone.0006598 19672310 PMC2720457

[22] Salamone J. D., Cousins M. S., McCullough L. D., Carriero D. L., & Berkowitz RJ. Nucleus accumbens dopamine release increases during instrumental lever pressing for food but not free food consumption. Pharmacol Biochem Behav. 1994;49(1):25–31. 7816884 10.1016/0091-3057(94)90452-9

[23] Treadway MT, Bossaller N, Shelton RC, Zald DH. Translational model of motivational anhedonia. J Abnorm Psychol. 2012;121(3):553–8. 10.1037/a0028813 22775583 PMC3730492

[24] Geaney JT, Treadway MT, Smillie LD. Trait anticipatory pleasure predicts effort expenditure for reward. 2015;10(6): e0131357 10.1371/journal.pone.0131357 26115223 PMC4482634

[25] Gard D. E., Gard M. G., Kring A. M., & John OP. Anticipatory and consummatory components of the experience of pleasure: a scale development study. J Res Pers. 2006;40(6):1086–102.

[26] Joiner TE, Brown JS, Metalsky GI. A test of the tripartite model’s prediction of anhedonia's specificity to depression: Patients with major depression versus patients with schizophrenia. Psychiatry Res. 2003;119(3):243–50. 12914895 10.1016/s0165-1781(03)00131-8

[27] Berridge KC, Robinson TE. What is the role of dopamine in reward: hedonic impact, reward learning, or incentive salience? Brain Res Rev. 1998;28(3):309–69. 9858756 10.1016/s0165-0173(98)00019-8

[28] Berridge KC, Robinson TE. Parsing reward. Trends Neurosci. 2003;26(9):507–13. 10.1016/S0166-2236(03)00233-9 12948663

[29] Watson D, Clark L a, Tellegen a. Development and validation of brief measures of positive and negative affect: the PANAS scales. J Pers Soc Psychol. 1988;54(6):1063–70. 3397865 10.1037//0022-3514.54.6.1063

[30] Bartoszek G. & Cervone D. Toward an implicit measure of emotions: Ratings of abstract images reveal distinct emotional states. Cogn Emot. 2016; 7:1–15. 10.1080/02699931.2016.1225004 27603515

[31] Fazio RH, Olson MA. Implicit measures in social cognition research: Their meaning and use. Annu Rev Psychol. 2003;54:297–327. 10.1146/annurev.psych.54.101601.145225 12172003

[32] Hardin J.W. and Hilbe JM. Generalized estimating equations Chapman & Hall; 2003.

[33] Wardle MC, Treadway MT, Mayo LM, Zald DH, de Wit H. Amping up effort: effects of d-amphetamine on human effort-based decision-making. J Neurosci. 2011;31(46):16597–602. 10.1523/JNEUROSCI.4387-11.2011 22090487 PMC3234999

[34] Tabachnick BG, Fidell LS. Using multivariate statistics. 6th editio. Pearson; 2012.

[35] Willner P, Muscat R, Papp M. Chronic mild stress-induced anhedonia: A realistic animal model of depression. Neurosci Biobehav Rev. 1992;16(4):525–34. 1480349 10.1016/s0149-7634(05)80194-0

[36] Admon R, Holsen LM, Aizley H, Remington A, Whitfield-Gabrieli S, Goldstein JM, et al Striatal hypersensitivity during stress in remitted individuals with recurrent depression. Biol Psychiatry. 2015;78(1):67–76. 10.1016/j.biopsych.2014.09.019 25483401 PMC4383718

[37] Treadway M. T., Peterman J. S., Zald D. H., & Park S. Impaired effort allocation in patients with schizophrenia. Schizophr Res. 2014; 161(2–3): 382–5. 10.1016/j.schres.2014.11.024 25487699 PMC4308548

[38] McClelland G. H., & Judd CM. Statistical difficulties of detecting interactions and moderator effects. Psychol Bull. 1993;114(2):376 8416037 10.1037/0033-2909.114.2.376

[39] Winer ES, Cervone D, Bryant J, McKinney C, Liu RT, Nadorff MR. Distinguishing mediational models and analyses in clinical psychology: Atemporal associations do not imply causation. J Clin Psychol. 2016; 72(9): 947–55. 10.1002/jclp.22298 27038095

[40] Bartoszek G., & Winer ES. Spider-fearful individuals hesitantly approach threat, whereas depressed individuals do not persistently approach reward. J Behav Ther Exp Psychiatry. 2015;46:1–7. 10.1016/j.jbtep.2014.07.012 25164091

[41] Pizzagalli D. A., Jahn A. L., O’Shea JP. Toward an objective characterization of an anhedonic phenotype: A signal-detection approach. Biol Psychiatry. 2005;57:319–27. 10.1016/j.biopsych.2004.11.026 15705346 PMC2447922

[42] Winer ES, Cervone D, Newman LS, Snodgrass M. Subchance perception: Anxious, non-defensive individuals identify subliminally-presented positive words at below-chance levels. Pers Individ Dif. 2011;51(8):996–1001.

